# Statistical power estimation dataset for external validation GoF tests on EVT distribution

**DOI:** 10.1016/j.dib.2019.104071

**Published:** 2019-05-28

**Authors:** Federico Reghenzani, Giuseppe Massari, Luca Santinelli, William Fornaciari

**Affiliations:** a DEIB, Politecnico di Milano, Milano, Italy; b DTIS, Onera, Toulouse, France

## Abstract

This paper presents the statistical power estimation of goodness-of-fit tests for Extreme Value Theory (EVT) distributions. The presented dataset provides quantitative information on the statistical power, in order to enable the sample size selection in external validation scenario. In particular, high precision estimations of the statistical power of KS, AD, and MAD goodness-of-fit tests have been computed using a Monte Carlo approach. The full raw dataset resulting from this analysis has been published as reference for future studies: https://doi.org/10.17632/hh2byrbbmf.1.

**Table:** Specification Table

Subject Area	Statistics
More specific subject area	Extreme Value Theory
Type of data	CSV text files, tables
How data was acquired	Monte Carlo approximation via CINECA supercomputing facility (Galileo cluster). The software has been developed in C++ over MPI/OpenMP frameworks.
Data format	Raw and aggregated
Experimental factors	The statistical testing procedures have been applied to synthetic time traces generated from known distributions. The process has been repeated several times collecting the test results.
Experimental features	The statistical tests results have been aggregated to obtain the statistical power estimation.
Data source location	CINECA, Segrate (MI), Italy
Data accessibility	Full raw dataset: https://doi.org/10.17632/hh2byrbbmf.1
	Data in aggregated form are presented in this paper.

**Table:** 

**Value of the data** • The dataset described in this paper provides an estimate of the statistical power of Goodness-Of-Fit (GoF) tests performed on Extreme Value distributions. • Several fields can benefit from the availability of this dataset, especially where it is necessary to select a proper sample size for the execution of GoF tests. • The statistical power data have been computed on a *case 0* scenario (also called *external validation*), i.e. when the samples used to perform the test are a different set with respect to the samples used to estimate the reference distribution. • The Monte Carlo approximation used to compute the statistical power has been performed on a very large number of sample ($10^{9}$) to guarantee a high level of accuracy of the results.

## 1 Data

The dataset described in this paper provides an estimate of the statistical power of Goodness-Of-Fit (GoF) tests. Its analytical calculation is in fact usually not easy: for most of GoF tests a closed form expression does not even exist. This estimation is necessary to properly select the sample size for testing procedures, thus reducing the type-II errors, i.e. the inability to reject the null hypothesis when it is actually false. The availability of this dataset can be advantageous for several fields, where the selection of the sample size is often performed with empirical procedures and where the results are often interpreted in a too optimistic view [1]. The GoF tests aim at identifying the deviation of data samples from a given distribution. However, if the test is not able to identify such null hypothesis violation, nothing can be stated and the statistical power becomes the only quantitative value that gives us the test result reliability information. The GoF tests have not been studied in *case 0* scenario (called also *external validation*) for EVT distributions, i.e. when the samples used to perform the test are a different set w.r.t. the samples used to estimate the reference distribution. In particular, to the best of our knowledge, quantitative information of only *case 3* scenarios is available in literature [2], while no *case 0* power analysis for such distribution classes is available in literature. This dataset wants exactly to fill this gap.

The statistical power computation has been performed with Monte Carlo approximations on a very large number of samples ($10^{9}$). This guaranteed a high level of accuracy of the results. This, together with the external validation scenario, is an interesting feature for recent applications of the EVT. One of the possible use-case of this dataset is *probabilistic real-time computing* [3], where EVT is used to estimate the probabilistic Worst-Case Execution Time (WCET) of the computer tasks. In this scenario, the confidence level of the statistical test is critical. A false-negative result may indeed lead to an under-estimation of the WCET, which may be unacceptable for the production system [4]. This is the reason why we decided to build the statistical power dataset with the highest possible accuracy, enabling the selection of suitable sample size and ensuring a sufficient test result reliability [5].

### 1.1 Hypothesis testing and statistical power

In hypothesis testing, the null hypothesis ($H_{0}$) is rejected when the observed data strongly suggest that it is false, in favour to an alternative hypothesis ($H_{1}$). On the contrary, if the null hypothesis cannot be rejected, nothing can be inferred about the truthfulness of any hypothesis. The **statistical power** is defined as the probability to incur in a *Type II error*, i.e. the failure to reject the null hypothesis when it is actually false. This concept can be expressed with the following conditional probability: $P\left(\text{not}\;\text{reject}\;H_{0}|H_{0}\;\text{is}\;\text{false}\right)$. This work presents the estimated statistical power of three Goodness-of-Fit (GoF) tests: Kolmogorov-Smirnov (KS) [6], Anderson-Darling (AD) [7], and Modified Anderson-Darling (MAD) [8] for EVT distributions. Other common tests have been excluded, for example the Chi-Squared (CS) and Cramer-von Mises (CvM) test, because state-of-the-art works already showed that they have a lower statistical power with respect to KS or AD [9], [10].

Regarding the specific EVT case, the work of Heo et al. [2] estimated the AD and MAD test critical values and power, by using a Montecarlo approach for GoF test of EVT distributions. The critical values were computed for a scenario where the model parameters to be tested were estimated from the same data used for the test. This scenario is commonly referred to as *Case 3*, i.e., the assumed distribution parameters are unknown. The *a priori* knowledge of the distribution parameters (*Case 0*) in fact, is not usually available for most of classical EVT applications. However, in some cases, e.g. the probabilistic real-time computing previously mentioned, we can easily increase the sample size, because getting new samples requires a low effort. For this reason, the *Case 0* can be applied, by drawing different independent samples for model parameter estimation and for model validation. This enables the possibility to perform the *external validation* that leads, in general, to the most stringent and unbiased test [11].

Generally, statistical power estimations for *Case 0* are not representative of *Case 3* and vice versa. This makes the data provided with this paper extremely valuable, because they represent a highly accurate estimation of the GoF statistical power for the external validation scenario and EVT distributions.

### 1.2 Statistical power estimation

The EVT distributions can be grouped under the Generalized Extreme Value distribution: GEV(μ,σ,ξ), where *μ* is the *location* parameter, *σ* is the *scale* parameter, and *ξ* is the *shape* parameter. The *location* and *scale* parameters determine the linear transformation of the standard GEV, while the *shape* parameter determines the distribution class. In this work, we explored all the three GEV classes as distribution references: a Gumbel distribution GEV(0,1,0), a Weibull distribution GEV(0,1,−0.5) and a Fréchet distribution GEV(0,1,0.5). For each of these distributions, the Goodness-of-Fit tests have been run on samples drawn from the other two GEV and from: a normal N(0,1), a t-student t(10), and a uniform distribution U(−2,3). The results for KS are shown in Table 1, for AD in Table 2, and for MAD in Table 3.

**Table 1:** Statistical powers of Kolmogorov-Smirnov (KS) test.

*G*_0_	*G*_1_	*α*	Sample size (*n*)									
			50	100	150	200	300	400	500	750	1000	2500
*GEV* (0, 1, 0)	*N* (0, 1)	0.05	0.433100925	0.883347765	0.991603951	0.999615010	0.999999874	1.000000000	1.000000000	1.000000000	1.000000000	1.000000000
		0.01	0.173221448	0.643452223	0.926426259	0.990668281	0.999969746	0.999999977	1.000000000	1.000000000	1.000000000	1.000000000
	*t* (10)	0.05	0.402221446	0.827773062	0.975704624	0.997320621	0.999988383	0.999999963	1.000000000	1.000000000	1.000000000	1.000000000
	*U* (–2, 3)	0.01	0.164124225	0.581214439	0.872639669	0.972233002	0.999500289	0.999996110	0.999999985	1.000000000	1.000000000	1.000000000
		0.05	0.286787074	0.754349990	0.962845802	0.996346524	0.999991250	0.999999992	1.000000000	1.000000000	1.000000000	1.000000000
	*GEV* (0, 1, −0.5)	0.01	0.092007617	0.442246778	0.782790629	0.944778594	0.999292443	0.999997245	0.999999994	1.000000000	1.000000000	1.000000000
		0.05	0.061924052	0.847865820	1.000000000	1.000000000	1.000000000	1.000000000	1.000000000	1.000000000	1.000000000	1.000000000
	*GEV* (0, 1, 0.5)	0.01	0.005506233	0.173621553	0.914148452	1.000000000	1.000000000	1.000000000	1.000000000	1.000000000	1.000000000	1.000000000
		0.05	0.109873147	0.293153608	0.549437525	0.740308878	0.943703112	0.991959149	0.999253784	0.999999586	1.000000000	1.000000000
		0.01	0.029933482	0.121708898	0.280459902	0.438755733	0.781235667	0.939418508	0.988237286	0.999932671	0.999999921	1.000000000

*GEV* (0, 1, 0.5)	*N* (0, 1)	0.05	0.869488165	0.999999315	1.000000000	1.000000000	1.000000000	1.000000000	1.000000000	1.000000000	1.000000000	1.000000000
		0.01	0.454885837	0.998873700	0.999999998	1.000000000	1.000000000	1.000000000	1.000000000	1.000000000	1.000000000	1.000000000
	*t* (10)	0.05	0.766801312	0.999765759	0.999999991	1.000000000	1.000000000	1.000000000	1.000000000	1.000000000	1.000000000	1.000000000
		0.01	0.359259013	0.983640502	0.999988015	0.999999997	1.000000000	1.000000000	1.000000000	1.000000000	1.000000000	1.000000000
	*U* (–2, 3)	0.05	0.367774702	1.000000000	1.000000000	1.000000000	1.000000000	1.000000000	1.000000000	1.000000000	1.000000000	1.000000000
		0.01	0.139806008	0.744566527	1.000000000	1.000000000	1.000000000	1.000000000	1.000000000	1.000000000	1.000000000	1.000000000
	*GEV* (0, 1, −0.5)	0.05	0.987657414	1.000000000	1.000000000	1.000000000	1.000000000	1.000000000	1.000000000	1.000000000	1.000000000	1.000000000
		0.01	0.632639500	1.000000000	1.000000000	1.000000000	1.000000000	1.000000000	1.000000000	1.000000000	1.000000000	1.000000000
	*GEV* (0, 1, 0)	0.05	0.032299787	0.231060818	0.576355557	0.852314165	0.995633829	0.999963685	0.999999914	1.000000000	1.000000000	1.000000000
		0.01	0.003184289	0.034069354	0.173820625	0.443183696	0.892633450	0.993613818	0.999882386	1.000000000	1.000000000	1.000000000

*GEV* (0, 1, −0.5)	*N* (0, 1)	0.05	0.284370451	0.629365918	0.862809163	0.953616572	0.996003172	0.999709102	0.999984209	0.999999990	1.000000000	1.000000000
		0.01	0.115952998	0.409541419	0.678458448	0.854765234	0.979383833	0.997758518	0.999791031	0.999999723	1.000000000	1.000000000
	*t* (10)	0.05	0.283091343	0.616658436	0.853660417	0.948713438	0.995402308	0.999673102	0.999984703	0.999999999	1.000000000	1.000000000
		0.01	0.116120515	0.399945876	0.664716864	0.844156028	0.976087763	0.997213016	0.999726448	0.999999688	0.999999999	1.000000000
	*U* (–2, 3)	0.05	0.826074656	0.998836102	0.999998678	1.000000000	1.000000000	1.000000000	1.000000000	1.000000000	1.000000000	1.000000000
		0.01	0.564242941	0.981799855	0.999913887	0.999999780	1.000000000	1.000000000	1.000000000	1.000000000	1.000000000	1.000000000
	*GEV* (0, 1, 0.5)	0.05	0.726868690	0.993646689	0.999951322	0.999999872	1.000000000	1.000000000	1.000000000	1.000000000	1.000000000	1.000000000
		0.01	0.466562747	0.954538684	0.999232900	0.999988813	1.000000000	1.000000000	1.000000000	1.000000000	1.000000000	1.000000000
	*GEV* (0, 1, 0)	0.05	0.200916378	0.658118197	0.907265288	0.983782467	0.999851447	0.999999511	0.999999999	1.000000000	1.000000000	1.000000000
		0.01	0.062618618	0.341107470	0.726574304	0.899661698	0.997334290	0.999960032	0.999999740	1.000000000	1.000000000	1.000000000

**Table 2:** Statistical powers of Anderson-Darling (AD) test.

*G*_0_	*G*_1_	*α*	Sample size (*n*)									
			50	100	150	200	300	400	500	750	1000	2500
*GEV* (0, 1, 0)	*N*(0, 1)	0.05	0.898883879	0.997852804	0.999984066	0.999999942	1.000000000	1.000000000	1.000000000	1.000000000	1.000000000	1.000000000
		0.01	0.718376305	0.980296191	0.999486072	0.999993816	1.000000000	1.000000000	1.000000000	1.000000000	1.000000000	1.000000000
	*t*(10)	0.05	0.900608464	0.996296207	0.999925665	0.999999076	1.000000000	1.000000000	1.000000000	1.000000000	1.000000000	1.000000000
		0.01	0.748572022	0.977738108	0.998990518	0.999971307	0.999999992	1.000000000	1.000000000	1.000000000	1.000000000	1.000000000
	*U*(–2, 3)	0.05	0.879039216	0.996647326	0.999962734	0.999999807	1.000000000	1.000000000	1.000000000	1.000000000	1.000000000	1.000000000
		0.01	0.637742192	0.966816380	0.998871526	0.999980674	0.999999999	1.000000000	1.000000000	1.000000000	1.000000000	1.000000000
	*GEV* (0, 1, −0.5)	0.05	0.506193505	0.988842459	0.999999819	1.000000000	1.000000000	1.000000000	1.000000000	1.000000000	1.000000000	1.000000000
		0.01	0.156587927	0.642128743	0.986360566	0.999993819	1.000000000	1.000000000	1.000000000	1.000000000	1.000000000	1.000000000
	*GEV* (0, 1, 0.5)	0.05	0.670656084	0.922479505	0.988032793	0.998722304	0.999993686	0.999999989	1.000000000	1.000000000	1.000000000	1.000000000
		0.01	0.459281936	0.781070668	0.936797739	0.986617002	0.999711112	0.999997335	0.999999992	1.000000000	1.000000000	1.000000000

*GEV* (0, 1, 0.5)	*N* (0, 1)	0.05	0.999748036	1.000000000	1.000000000	1.000000000	1.000000000	1.000000000	1.000000000	1.000000000	1.000000000	1.000000000
		0.01	0.995080617	0.999999963	1.000000000	1.000000000	1.000000000	1.000000000	1.000000000	1.000000000	1.000000000	1.000000000
	*t* (10)	0.05	0.999535626	1.000000000	1.000000000	1.000000000	1.000000000	1.000000000	1.000000000	1.000000000	1.000000000	1.000000000
		0.01	0.996014553	0.999999766	1.000000000	1.000000000	1.000000000	1.000000000	1.000000000	1.000000000	1.000000000	1.000000000
	*U* (−2, 3)	0.05	0.998987729	0.999999982	1.000000000	1.000000000	1.000000000	1.000000000	1.000000000	1.000000000	1.000000000	1.000000000
		0.01	0.994605043	0.999997776	1.000000000	1.000000000	1.000000000	1.000000000	1.000000000	1.000000000	1.000000000	1.000000000
	*GEV* (0, 1, −0.5)	0.05	0.999824564	1.000000000	1.000000000	1.000000000	1.000000000	1.000000000	1.000000000	1.000000000	1.000000000	1.000000000
		0.01	0.950973931	1.000000000	1.000000000	1.000000000	1.000000000	1.000000000	1.000000000	1.000000000	1.000000000	1.000000000
	*GEV* (0, 1, 0)	0.05	0.496070245	0.864390928	0.985947394	0.999396436	0.999999874	1.000000000	1.000000000	1.000000000	1.000000000	1.000000000
		0.01	0.268355771	0.570821100	0.843710853	0.968703009	0.999817934	0.999999875	1.000000000	1.000000000	1.000000000	1.000000000

*GEV* (0, 1,–0.5)	*N* (0, 1)	0.05	0.883425766	0.990317893	0.999349700	0.999962533	0.999999898	1.000000000	1.000000000	1.000000000	1.000000000	1.000000000
		0.01	0.806435001	0.975341606	0.997569908	0.999802265	0.999999137	0.999999998	1.000000000	1.000000000	1.000000000	1.000000000
	*t* (10)	0.05	0.953012799	0.998489273	0.999961459	0.999999177	1.000000000	1.000000000	1.000000000	1.000000000	1.000000000	1.000000000
		0.01	0.916712052	0.995729267	0.999835355	0.999994869	0.999999994	1.000000000	1.000000000	1.000000000	1.000000000	1.000000000
	*U* (−2, 3)	0.05	0.999998030	1.000000000	1.000000000	1.000000000	1.000000000	1.000000000	1.000000000	1.000000000	1.000000000	1.000000000
		0.01	0.999994727	1.000000000	1.000000000	1.000000000	1.000000000	1.000000000	1.000000000	1.000000000	1.000000000	1.000000000
	*GEV* (0, 1, 0.5)	0.05	0.999997678	1.000000000	1.000000000	1.000000000	1.000000000	1.000000000	1.000000000	1.000000000	1.000000000	1.000000000
		0.01	0.999996758	1.000000000	1.000000000	1.000000000	1.000000000	1.000000000	1.000000000	1.000000000	1.000000000	1.000000000
	*GEV* (0, 1, 0)	0.05	0.998992782	0.999998975	0.999999999	1.000000000	1.000000000	1.000000000	1.000000000	1.000000000	1.000000000	1.000000000
		0.01	0.998880701	0.999998793	1.000000000	1.000000000	1.000000000	1.000000000	1.000000000	1.000000000	1.000000000	1.000000000

**Table 3:** Statistical powers of Modified Anderson-Darling (MAD) test.

*G*_0_	*G*_1_	*α*	Sample size (*n*)									
			50	100	150	200	300	400	500	750	1000	2500
*GEV* (0, 1, 0)	*N* (0, 1)	0.05	0.867320643	0.997898948	0.999990214	0.999999977	1.000000000	1.000000000	1.000000000	1.000000000	1.000000000	1.000000000
		0.01	0.588673674	0.973245243	0.999478267	0.999995867	1.000000000	1.000000000	1.000000000	1.000000000	1.000000000	1.000000000
	*t* (10)	0.05	0.781941641	0.985645303	0.999484369	0.999987393	0.999999994	1.000000000	1.000000000	1.000000000	1.000000000	1.000000000
		0.01	0.491534388	0.919403834	0.993532836	0.999678517	0.999999668	1.000000000	1.000000000	1.000000000	1.000000000	1.000000000
	*U* (–2, 3)	0.05	0.435062367	0.842316752	0.981577615	0.999015458	0.999999594	1.000000000	1.000000000	1.000000000	1.000000000	1.000000000
		0.01	0.165284527	0.485703819	0.811172107	0.962024230	0.999674018	0.999999555	1.000000000	1.000000000	1.000000000	1.000000000
	*GEV* (0, 1, −0:5)	0.05	0.645973895	0.999995654	1.000000000	1.000000000	1.000000000	1.000000000	1.000000000	1.000000000	1.000000000	1.000000000
		0.01	0.109046892	0.906790997	0.999997798	1.000000000	1.000000000	1.000000000	1.000000000	1.000000000	1.000000000	1.000000000
	*GEV* (0, 1, 0:5)	0.05	0.791838371	0.961675361	0.994629272	0.999381894	0.999994651	0.999999961	1.000000000	1.000000000	1.000000000	1.000000000
		0.01	0.631781428	0.893099447	0.976268016	0.995786651	0.999913894	0.999998846	0.999999990	1.000000000	1.000000000	1.000000000

*GEV* (0, 1, 0:5)	*N* (0, 1)	0.05	0.999736838	1.000000000	1.000000000	1.000000000	1.000000000	1.000000000	1.000000000	1.000000000	1.000000000	1.000000000
		0.01	0.965085315	0.999999991	1.000000000	1.000000000	1.000000000	1.000000000	1.000000000	1.000000000	1.000000000	1.000000000
	*t* (10)	0.05	0.996004168	0.999999998	1.000000000	1.000000000	1.000000000	1.000000000	1.000000000	1.000000000	1.000000000	1.000000000
		0.01	0.914097219	0.999992894	1.000000000	1.000000000	1.000000000	1.000000000	1.000000000	1.000000000	1.000000000	1.000000000
	*U* (−2, 3)	0.05	0.559739086	0.999999974	1.000000000	1.000000000	1.000000000	1.000000000	1.000000000	1.000000000	1.000000000	1.000000000
		0.01	0.085883847	0.848460957	0.999999946	1.000000000	1.000000000	1.000000000	1.000000000	1.000000000	1.000000000	1.000000000
	*GEV* (0, 1, −0:5)	0.05	1.000000000	1.000000000	1.000000000	1.000000000	1.000000000	1.000000000	1.000000000	1.000000000	1.000000000	1.000000000
		0.01	0.948498429	1.000000000	1.000000000	1.000000000	1.000000000	1.000000000	1.000000000	1.000000000	1.000000000	1.000000000
	*GEV* (0, 1, 0)	0.05	0.269985358	0.803816020	0.984632476	0.999564876	0.999999972	1.000000000	1.000000000	1.000000000	1.000000000	1.000000000
		0.01	0.045445362	0.315514515	0.745273839	0.958954776	0.999873523	0.999999965	1.000000000	1.000000000	1.000000000	1.000000000

*GEV* (0, 1, −0:5)	*N* (0, 1)	0.05	0.861794911	0.987006580	0.999023454	0.999937794	0.999999803	1.000000000	1.000000000	1.000000000	1.000000000	1.000000000
		0.01	0.774748242	0.966673086	0.996200975	0.999643427	0.999997890	0.999999994	1.000000000	1.000000000	1.000000000	1.000000000
	*t* (10)	0.05	0.938597184	0.997569575	0.999925765	0.999998185	0.999999999	1.000000000	1.000000000	1.000000000	1.000000000	1.000000000
		0.01	0.895361187	0.993189832	0.999670695	0.999987242	0.999999992	1.000000000	1.000000000	1.000000000	1.000000000	1.000000000
	*U* (−2, 3)	0.05	0.999996144	1.000000000	1.000000000	1.000000000	1.000000000	1.000000000	1.000000000	1.000000000	1.000000000	1.000000000
		0.01	0.999991056	1.000000000	1.000000000	1.000000000	1.000000000	1.000000000	1.000000000	1.000000000	1.000000000	1.000000000
	*GEV* (0, 1, 0:5)	0.05	0.999997506	1.000000000	1.000000000	1.000000000	1.000000000	1.000000000	1.000000000	1.000000000	1.000000000	1.000000000
		0.01	0.999996636	1.000000000	1.000000000	1.000000000	1.000000000	1.000000000	1.000000000	1.000000000	1.000000000	1.000000000
	*GEV* (0, 1, 0)	0.05	0.999028457	0.999998993	1.000000000	1.000000000	1.000000000	1.000000000	1.000000000	1.000000000	1.000000000	1.000000000
		0.01	0.998893433	0.999998740	0.999999995	1.000000000	1.000000000	1.000000000	1.000000000	1.000000000	1.000000000	1.000000000

### 1.3 Sensitivity analysis

**Fig. 1:**
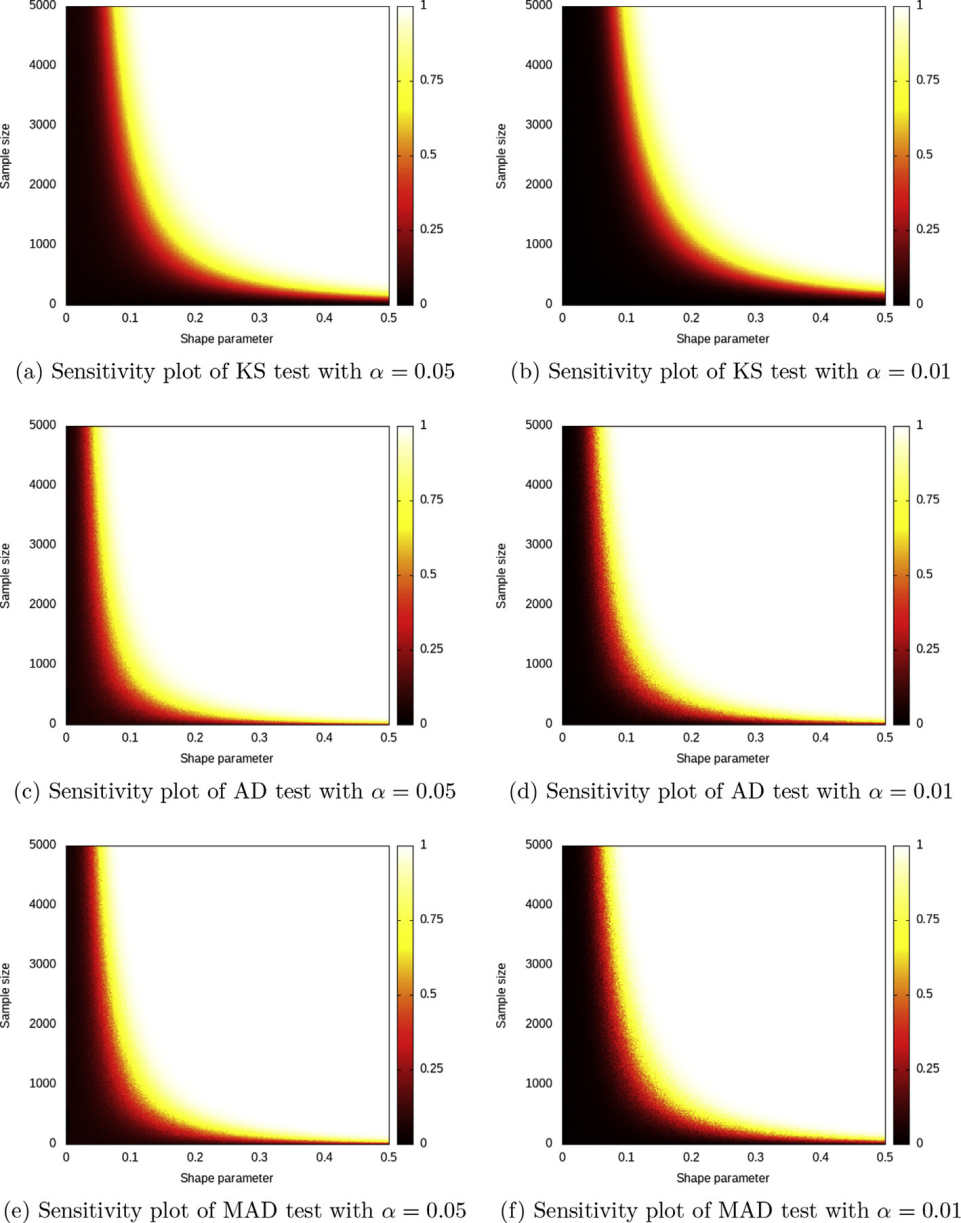
Sensitivity plots for $G_{0}∼GEV\left(0,1,0\right)$.$G_{1}∼GEV\left(0,1,0.5\right)$

Given the statistical power results of the representative test cases, we performed a sensitivity analysis on the sample size and the shape parameter *ξ* of the GEV distribution. The results are depicted in Fig. 1, while the raw data are available in the dataset.

## 2 Experimental design, materials, and methods

The analytical computation of the statistical power, and consequently the selection of the appropriate sample size, is usually not possible, due to the frequent lack of the *effect size knowledge*, i.e. the real characterization of the population's distribution from which the samples have been collected. Consequently, Munthen et al. [12] studied the usage of Monte Carlo methods to select the sample size and determine the testing power. To this purpose, we need to define a set of tuples representing the test conditions. In particular, the Monte Carlo sampling is executed for every tuple $\left(D,n,\alpha ,G_{1},G_{2}\right)$, where *D* is the statistic of the test under analysis, *n* is the sample size, *α* the level of significance, $G_{1},G_{2}$ are respectively the reference distribution with cumulative distribution function F(x) and the empirical distribution with cumulative distribution function $F_{n}\left(x\right)$.

The statistics *D* for KS, AD and MAD test can be computed using their discretized forms [13], [14], [15]:

$$D_{KS}=\underset{x}{\text{sup}}|F_{n}\left(x\right)-F\left(x\right)|$$

$$D_{A^{2}}=-n-\frac{1}{n}\overset{n}{\underset{i=1}{\sum }}\left(2i-1\right)\text{log}\left(F\left(x_{i}\right)\right)-\frac{1}{n}\overset{n}{\underset{i=1}{\sum }}\left(2n-2i+1\right)\text{log}\left(F\left(1-x_{i}\right)\right)$$

$$D_{AU^{2}}=\frac{n}{2}-2\overset{n}{\underset{i=1}{\sum }}F\left(x_{i}\right)-\overset{n}{\underset{i=1}{\sum }}\frac{2n-2i+1}{n}\text{log}\left(F\left(1-x_{i}\right)\right)$$

The critical values (line 8) are computed with the following closed form – valid for n>30 – for KS test [16]:

$$\text{critical}\_{\text{value}}_{KS}=\frac{\sqrt{-\frac{1}{2}\text{log}\frac{\alpha }{2}}}{\sqrt{n}}$$

Instead, for (M)AD test no closed form is available because the critical value computation procedure strongly depends on $G_{0}$. We performed a dedicated Monte Carlo estimation similar to the method used by Heo et al. [2] to get (M)AD critical values. To double check, the resulting values have been used in the statistic comparison against data coming from $G_{0}$ (i.e. when $H_{0}$ is true) and the tests failed to not reject $H_{0}$ with *α* probability, as expected by the definition of significance level.

Algorithm 1 Power estimation with Monte Carlo simulations.

**Figure:**
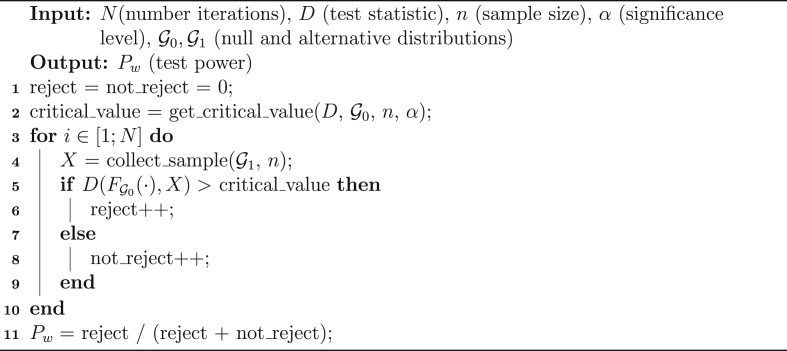

The estimation algorithm is shown in Listing 2. For each scenario, the critical value is computed (line 2) and a large number of explorations *N* is performed (lines 3–10). Each time, we draw a sample from the reference distribution (line 4) and we check if the statistic *D* of the ecdf matches or not with the drawn sample, comparing it with the critical value (line 5). If the statistic value is higher than the critical value, then the sample is rejected (line 6), otherwise not (line 8). Finally, the ratio rejection over total samples provide us the statistical power (line 11). If the test is able to detect the differences between $G_{1}$ and $G_{2}$ we expect to get a value near 1 for this ratio. In this specific Monte Carlo simulation, the standard error of power can be computed as [17]:

(1) $$\sqrt{\frac{R\left(N-R\right)}{N^{3}}}$$

where R≤N is the number of rejects (the accumulation variable of line 12). The standard error is decreasing when N→∞ and when R→N, i.e. when statistical power approaches the maximum value 1.

The selected values for parameters of each Monte Carlo estimation are:

• $N=10^{9}$: number of Monte Carlo iterations;
• *D*: the test statistics previously described;
• *n*: the sample size. Exploring all the possible values would have increased in a non-sustainable way the computational effort required by the Monte Carlo simulations. Since the power test function is a non-decreasing function of *n*, we explored them easily selecting the following values: n=(50,100,150,200,300,400,500,750,1000,2500);
• *α*: the significance level. We studied the traditional values of 0.05 and 0.01.

The simulations ran on 4 nodes of CINECA supercomputing facility (GALILEO-A1 cluster, 2 x Intel Xeon E5-2697v4@2.3GHz per node) for a total of 144 CPU cores. It took ≈ 13h for KS tests, ≈ 17.5h for AD test, ≈ 16h for MAD test.

Given the statistical power results of the representative test cases, we performed a sensitivity analysis on sample size *n* and shape parameter *ξ*. The power was obtained by using the same procedure of Algorithm 2, but reducing considerably the number of iterations *N*, in order to enable a fine-grain analysis with a sustainable computational effort. By exploring the integer sample size space and the real shape parameter space, the Monte Carlo simulations carry out a power matrix of sizes $\bar{\bar{\xi }}\times \bar{\bar{n}}$ (where $\bar{\bar{\cdot }}$ is the cardinality of the set of all the possible values of ⋅).
